# The effectiveness of a protocol without routine radiographs for follow-up of adolescent idiopathic scoliosis patients (CURVE): a study protocol

**DOI:** 10.2340/17453674.2024.40904

**Published:** 2024-06-17

**Authors:** Jurre T F BAETSEN, Miranda L VAN HOOFF, Pepijn BISSELING, Johanna M VAN DONGEN, Dineke G VAN DE FLIERT, Eric HOEBINK, Diederik H R KEMPEN, Joost P H J RUTGES, Tom P C SCHLÖSSER, Hanneke M VAN WEST, Philip J VAN DER WEES, Paul C WILLEMS, MARINUS DE KLEUVER, on behalf of Dutch AIS consortium

**Affiliations:** 1 Department of Orthopedic Surgery, Radboud University Medical Center, Nijmegen; 2 Department of Orthopedic Surgery, Sint Maartenskliniek, Nijmegen; 3 Vrije Universiteit Amsterdam, Department of Health Sciences, Faculty of Science, Amsterdam Movement Science Research Institute, Amsterdam; 4 Department of Health Sciences, Faculty of Science, Vrije Universiteit Amsterdam, Amsterdam Public Health Research Institute, Amsterdam; 5 Department of Orthopedic Surgery, Amphia, Breda; 6 Department of Orthopedic Surgery, OLVG, Amsterdam; 7 Department of Orthopedic Surgery, Erasmus Medical Center, Rotterdam; 8 Department of Orthopedic Surgery, University Medical Center Utrecht, Utrecht; 9 IQ Healthcare and Department of Rehabilitation, Radboud University Medical Center, Nijmegen; 10 Department of Orthopedic Surgery, Maastricht University Medical Center, Maastricht, The Netherlands; 11 Dutch AIS consortium: E Heerdt, M van den Boogaart, E Jacobs, P van Hugten, Department of Orthopedic Surgery, Maastricht University Medical Center, Maastricht; L W van Rhijn, M C Kruyt, Department of Orthopedic Surgery, University Medical Center Utrecht, Utrecht; J Renkens, F Joor, M Reijman, S Pols, H, Olderkamp, Department of Orthopedic Surgery, Erasmus MC, Rotterdam; P Horsting, D Schrander, L de Klerk, T Stevens Department of Orthopedic Surgery,, Sint Maartenskliniek, Nijmegen; M C Altena, M L M van der Hulst, A J Rasker, Department of Orthopedic Surgery, OLVG, Amsterdam; M Nieuwenhuijse, J de Haan, I Wiljouw, Department of Orthopedic Surgery, Amphia, Breda; B J van Royen, A Stadhouder, Department of Orthopedic Surgery, Amsterdam UMC, Amsterdam; A K Mostert, M P Cornelissen, R Munnik, G B van Solinge, D R de Boer, Department of Orthopedic Surgery, Isala, Zwolle; C Faber, G J F J Bos, F H Wapstra, Department of Orthopedic Surgery, UMCG, Groningen; A Spoor, R Geuze, S Bossers, Department of Orthopedic Surgery, Elisabeth Twee steden Ziekenhuis, Tilburg; H Graat, K Slot, E J Meurs, J L Benner, P P A Vergroesen, W J Pluymakers, Department of Orthopedic Surgery, Noordwest Ziekenhuisgroep, Alkmaar; E Kraaneveld, A Stadhouder, Department of Orthopedic Surgery, Dijklander, Hoorn; P Haen, Department of Orthopedic Surgery, Groene Hart Ziekenhuis, Gouda; W J Marijnissen, Department of Orthopedic Surgery, Albert Schweitzer Ziekenhuis, Dordrecht; R Hoogendoorn, K Ottink, M van der Poel, Department of Orthopedic Surgery, Spaarne Gasthuis, Hoofddorp; P B de Witte, Department of Orthopedic Surgery, LUMC, Leiden; H Sonneveld, T D Berendes, Meander M C, Department of Orthopedic Surgery, Amersfoort; J Nieuwenhuis, Department of Orthopedic Surgery, VieCuri, Venlo; B J Blom, R Zwiers, Department of Orthopedic Surgery, Flevo ziekenhuis, Almere; J van Susante, L de Jong, Department of Orthopedic Surgery, Rijnstate, Arnhem; WM Bakker, Department of Orthopedic Surgery, Maasstad ziekenhuis, Rotterdam; P G Lammers, W A Meijwaard, Department of Orthopedic Surgery, Sint Jansdal, Hardewijk; J H van Linge, Department of Orthopedic Surgery, Juliana kinderziekenhuis, Den Haag; GH Moens, Department of Orthopedic Surgery, Bernhoven, Uden; Y M den Hartog, Department of Orthopedic Surgery, Medisch Spectrum Twente, Enschede; J Lutjeboer, Department of Orthopedic Surgery, Admiraal de Ruyter ziekenhuis, Goes

## Abstract

**Background and purpose:**

Current follow-up protocols for adolescent idiopathic scoliosis (AIS) are based on consensus and consist of regular full-spine radiographs to monitor curve progression and surgical complications. Consensus exists to avoid inappropriate use of radiographs in children. It is unknown whether a standard radiologic follow-up (S-FU) approach is necessary or if a patient-empowered follow-up (PE-FU) approach can reduce the number of radiographs without treatment consequences.

**Methods and analyses:**

A nationwide multicenter pragmatic randomized preference trial was designed for 3 follow-up subgroups (pre-treatment, post-brace, post-surgery) to compare PE-FU and S-FU. 812 patients with AIS (age 10–18 years) will be included in the randomized trial or preference cohorts. Primary outcome is the proportion of radiographs with a treatment consequence for each subgroup. Secondary outcomes consist of the proportion of patients with delayed initiation of treatment due to non-routine radiographic follow-up, radiation exposure, societal costs, positive predictive value, and interrelation of clinical assessment, quality of life, and parameters for initiation of treatment during follow-up. Outcomes will be analyzed using linear mixed-effects models, adjusted for relevant baseline covariates, and are based on intention-to-treat principle. Study summary: (i) a national, multicenter pragmatic randomized trial addressing the optimal frequency of radiographic follow-up in patients with AIS; (ii) first study that includes patient-empowered follow-up; (iii) an inclusive study with 3 follow-up subgroups and few exclusion criteria representative for clinical reality; (iv) preference cohorts alongside to amplify generalizability; (v) first study conducting an economic evaluation comparing both follow-up approaches.

Adolescent idiopathic scoliosis (AIS) is a 3-dimensional deformity of the spine and trunk in adolescents that occurs in 2–3% of healthy children [1]. Because severe curves have a negative impact on quality of life in adulthood, patients with AIS are regularly seen for radiographic follow-up to detect curve progression, so that timely treatment can be initiated [2]. Treatment consists of thoracolumbar orthosis (brace) or surgery. The length and intensity of radiographic follow-up after treatment varies widely among physicians and lack of consensus exists on the need for long-term radiographic follow-up.

The current standard of care is based on regular radiographic follow-up as recommended in national and international consensus papers [3-5]. Regular radiographic follow-up leads to multiple radiographs without consequences for individual patient treatment. The developing tissues of adolescent patients are exposed to ionizing radiation, which previously has been associated with an increase in risk of breast and endometrial cancer [6,7]. Although technological advances have reduced the radiation dose tremendously, lack of evidence to support routine radiographic follow-up still warrants investigation of new protocols [8,9]. Additionally, a reduction of radiographs reduces the financial burden on the healthcare system. To optimize follow-up protocols of patients with AIS, an AIS Consortium including Dutch scoliosis treatment centers was established to evaluate a new patient-empowered follow-up protocol.

The purpose of this nationwide study is to evaluate whether a reduction in the number of radiographs can be achieved without negative consequences for individual treatment of patients with AIS, by combining clinical measurements and empowering patients through education and self-management.

The hypothesis is that the patient-empowered follow-up-protocol results in a (cost-)effective and safe reduction of the number of radiographs.

## Methods and analysis

### Study design and setting

**Figure 1:**
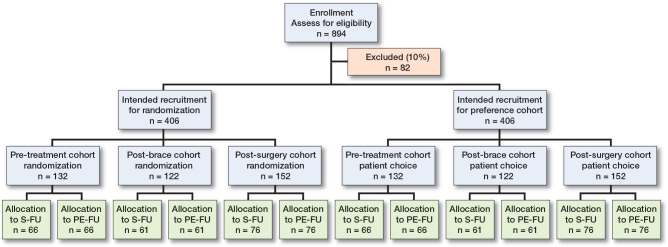
CONSORT flow of patients throughout the CURVE study.

A multicenter pragmatic trial, a partially randomized preference trial (PRPT), will be performed in The Netherlands. Dutch scoliosis treatment centers were asked to participate in this study. The PRPT consists of 3 separate randomized controlled trials (RCTs), including patients with AIS who are followed in the pre-treatment, post-brace, or postoperative phase (i.e., subgroups). Alongside the RCTs, prospective patient preference cohorts are included for each follow-up subgroup. In these cohorts, all patients are monitored who are not willing to be randomized to either follow-up arm, but who are still willing to participate in the study and who prefer either follow-up protocol. The follow-up period is 24 months per patient. A flow of the study progress following the CONSORT statement [10] is provided in Figure 1. The study will be reported according to CONSORT guidelines.

### Surgeon expertise

Orthopedic spine surgeons participating in the PRPT have to be competent in both follow-up-protocols. Before local study initiation, all participating local study personnel are obliged to complete an online training module, including questionnaires, in which the study-specific protocols and related procedures are evaluated. Residents can participate under the attending supervising surgeon.

### Patient selection

#### Eligibility

To be eligible for this study, a patient must meet the selection criteria for one of the follow-up subgroups as listed in Table 1 (subgroups: pre-treatment, post-brace, post-surgery).

**Table 1:** Inclusion and exclusion criteria

**General inclusion criteria**
Patients with adolescent idiopathic scoliosis (AIS). Patients scheduled for follow-up in 1 of the participating centers. Biplanar (posterior-anterior [PA] and lateral) full-spine radiographs within an acceptable period from inclusion. Understanding of the Dutch language. Signed informed consent.
**General exclusion criteria**
Patients with juvenile or infantile idiopathic scoliosis with the diagnosis of onset under the age of 10. Patients who are undergoing brace treatment. Patients who have undergone previous spinal surgery and are undergoing revision surgery.
**Specific inclusion criteria pre-treatment group**
Girls aged ≤ 14 years (i.e., 10–14 years) and boys ≤ 15 years (i.e., 10–15 years). These patients generally have 2 years of remaining skeletal growth [22]. Girls: pre-menarche up to 6 months post-menarche (to estimate end of growth). A primary coronal curve with a Cobb angle of 10–25°. *Note on growth:* to validate the inclusion, growth velocity will be determined to avoid stability of growth (threshold: < 0.4 cm/year [23]). Stability in growth in this study is defined as < 0.2 cm/6 months. At baseline and at 6 months’ follow-up assessment height (cm) will be determined. Growth velocity, i.e., height difference over 6 months, will be calculated. After 1 year the treatment validity of inclusion will be determined and patients who are stabilized will be replaced.
**Specific exclusion criteria pre-treatment group**
Skeletally mature patients.
**Specific inclusion criteria post-brace group**
Patients aged 12–18 years.
Within 3 months after termination of brace treatment.
Minimum of 6 months of brace treatment.
**Specific inclusion criteria post-surgery group**
Patients aged 12–18 years.

#### Recruitment and informed consent

Eligible patients are informed and invited to participate by their local treating orthopedic surgeon. To obtain informed consent, eligible patients and their parents will be handed an age-specific patient information form (PIF). According to Dutch regulations, for each subgroup different PIFs are developed for each age category (10–11, 12–15, and > 15 years of age). The first patients were included in July 2022.

### Randomization and blinding

After obtaining informed consent, patients will either be randomized or allocated to the protocol of their preference. Randomization is performed according to a minimization algorithm in a 1:1 ratio to either the standard follow-up protocol (S-FU) or the patient-empowered follow-up protocol (PE-FU). Randomization will be performed in CASTOR EDC (www.castoredc.com), an online secured study and data-management system with built-in randomization (random permuted block size 2:4). Considering the nature of this project, blinding to the follow-up allocation for patients and medical staff is not possible. The trial statistician will be blinded for the follow-up allocation.

### Study interventions

#### Patient-empowered follow-up (PE-FU) protocol (Figure 2)

The PE-FU protocol consists of patient-reported outcome measures (PROMs), the Scolioscope and clinical assessment including physical examination by the physician. The Scolioscope is a self-assessment tool that measures the trunk rotation, similar to what is measured with the Bunnell scoliometer (Figure 3). At baseline and at last follow-up (24 months) biplanar radiographs are taken. Instead of routine radiographs, at all intervening follow-up visits radiographs will only be taken when curve progression or postoperative complications are suspected based on so-called “sense of alarm” criteria, which is a signal for the treating physician to consider whether a radiograph is appropriate.

**Figure 2:**
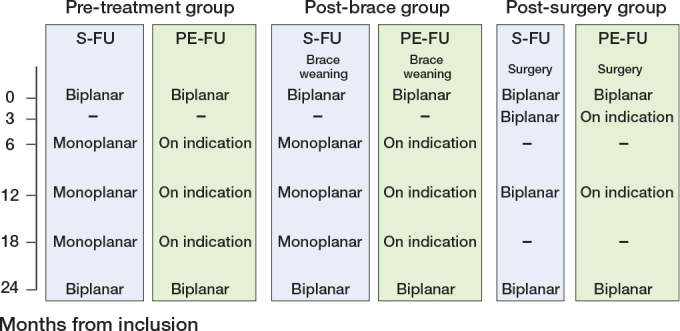
Follow-up scheme for each of the 3 predefined subgroups of the current study with timeline, with time in months from inclusion to radiography, monoplanar (posterior-anterior), or biplanar. S-FU = standard follow-up, PE-FU = patient empowered follow-up.

**Figure 3:**
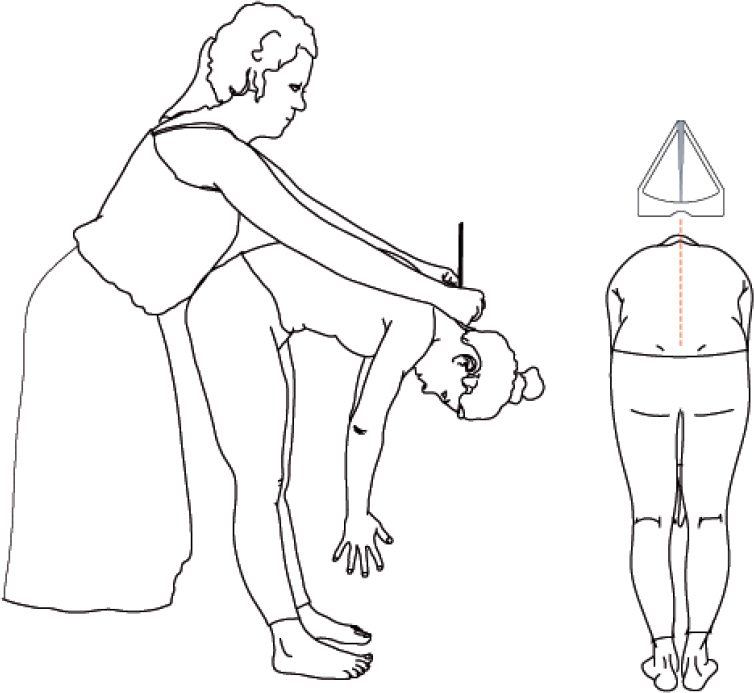
Angle of trunk rotation measurement using the Scolioscope. © Productzaken

“Sense of alarm” is based on any concern by the orthopedic surgeon, patient, or parent that warrants a radiograph and any deterioration on the PROMs, Scolioscope, and clinical assessments. For both the Bunnell scoliometer and the Scolioscope a threshold of respectively ≥ 4° and ≥ 4 points increase will be used [11]. No known thresholds exist for the included PROMs to support the decision to take a radiograph; the clinical view of the physician is important and included. For all non-protocolized radiographs taken, the reason is reported.

#### Standard follow-up (S-FU) protocol (Figure 2)

The S-FU protocol consists of routine full spine radiographs taken at inclusion and each follow-up visit, to detect possible curve progression or to rule out postoperative complications. For the post-surgical group, biplanar (posterior-anterior [PA] and lateral) radiographs are taken at each follow-up assessment. For pre-treatment and post-brace groups uniplanar radiographs are taken, with biplanar radiographs at baseline and at last follow-up (24 months) visit.

### Patient characteristics and outcome measures (Table 2)

At baseline, relevant patient characteristics are collected to describe the study sample, including the 3 different subgroups. Primary outcome is defined as the proportion of radiographs that has led to treatment consequences in each subgroup (pre-treatment, post-brace, post-surgery). A treatment consequence is defined as start of brace therapy or any surgical intervention. Secondary outcome measures include both patient and clinician-reported outcomes.

**Table 2:** Baseline characteristics, secondary outcome measures, and measurement moments

Variable	Month of follow-up assessments					
	Baseline	3	6	12	18	24
Post-surgery group	√	√	–	√	–	√
Pre-treatment group and post-brace group	√	–	√	√	√	√
**Baseline patient characteristics**						
Patient-reported and clinician-reported.	√	–	–	–	–	–
Including age, sex, family origin (CBS [24]), BMI, concomitant diseases, concomitant medication, family history of AIS, sports, education status						
**Secondary outcome measures**						
Health-related quality of life (PROM): EuroQol-5						
Dimensions (EQ-5D-5L – Dutch version [25]).	√	√	√	√	√	√
A 5-item questionnaire, consisting of 5 dimensions (mobility, self-care, usual activities, pain/discomfort, and anxiety/depression) QALYs will be estimated using the Dutch tariff [25]						
Condition-specific quality of life (PROM):						
SRS-22r – Dutch version [26]	√	√	√	√	√	√
A 22-item questionnaire, consisting of 5 domains (pain [5 items], self-image [5 items], function ([5 items], mental health [5 items], and satisfaction with management [2 items]) and a total score, with scores ranging 1 to 5 for each item						
Pain (PROM) Numerical Rating Scale (NRS)	√	√	√	√	√	√
Ranging from 0 (no pain) to 10 (worst imaginable pain) for mean pain since last visit						
Spinal appearance questionnaire (PROM): Short SAQ	√	√	√	√	√	√
A 14-item questionnaire [27,28], consisting of a total score and 2 domain scales, “appearance” (10 items) and “expectations” (4 items), with scores ranging from 1 to 5						
Global Perceived Effect (PROM): GPE	√	√	√	√	√	√
2 items ranging in score from 1 to 7 regarding satisfaction with the treatment effect [29]						
Education status (PROM) Patient-reported	√	√	√	√	√	√
Absence from school, absence from physical education (PE) classes, frequency of missing school exams, necessity to repeat a class, support and alternatives provided by the school						
Health-related and societal costs (PROM): Questionnaires	√	√	√	√	√	√
Costs will be collected with cost questionnaires (modified iPCQc , iMCQ, and iViCQ). Resource use will be valued in accordance with the Dutch Manual for Costing Studies in Health Care [30]						
Angle of trunk rotation (ATR, °; patient-reported)						
Scolioscope (only PE-FU)	√	√	√	√	√	√
The asymmetry of the spine results in an ATR and a rib hump, which will be measured by the parent/caregiver						
Curve progression (ATR, °; clinician-reported)						
The Bunnell scoliometer [11]	√	√	√	√	√	√
Patient-reported questions after surgery (post-surgery group only): Return to school, return to sport	√	√	–	√	–	√
Radiographic assessment (full spineradiograph; clinician-reported)	√	√	√	√	√	√
Progression of deformity in the primary and compensatory Cobb angels (°).						
Biplanar radiographs for post-surgery group and at baseline and 24 months for pre-treatment and post-brace groups. According to S-FU monoplanar radiographs are taken during follow-up visits for pre-treatment and post-brace groups						
Radiation exposure:						
Calculation using reference values	√	√	√	√	√	√
Assessment (serious) adverse events (clinician-reported)	–	√	√	√	√	√

**Table 2:** Abbreviations

PROM = patient reported outcome measure. iMTA = institute for Medical Technology Assessment. iPCQ = iMTA Productivity Cost Questionnaire. iMCQ = iMTA Medical Consumption Questionnaire. iViCQ = iMTA Valuation of Informal Care Questionnaire PE-FU = patient-empowered follow-up protocol. S-FU = standard follow-up protocol.

### Study follow-up and data collection

The primary and the secondary outcomes will be assessed at all follow-up visits (Table 2). Baseline patient characteristics and outcome data (over time) are collected in an online electronic case report form (eCRF). The eCRF that will be used is KLIK (Dutch acronym for Quality of Life in Clinical Practice [12]) (www.hetklikt.nu). KLIK is an online PROM tool made for children and accessible for patients and clinicians during consultation to evaluate any sense of alarm according to the clinical criteria or concern of the patient/parent.

### Sample size

The sample size is based on identifying superiority of the PE-FU in each individual RCT. The 3 subgroups for this study differ in nature; this means that separate sample size calculations are required. An unpublished survey of Dutch patients with AIS showed that 50% of patients had a preference for 1 of the 2 cohorts (the preference cohorts) and 50% were willing to participate in the RCT, which is comparable to an American study that used a similar design [13]. By doubling the sample size needed for the RCTs, the total sample size is achieved. As no previous studies regarding this subject are available, the literature was searched for patient samples with AIS that resemble the study population and that showed progression of the curve during natural history [14] or after surgery [15]. Assumptions were made based on the literature and expert opinion. Typically, patients with AIS and their parents/caregivers are very compliant with follow-up. A 10% dropout or lost-to-follow-up for the 3 subgroups is expected. Based on the above, for this study a total of 812 patiens is required (alpha 0.05; beta 0.80). This means for the RCT: 66 per arm for pre-treatment, 61 per arm for post-brace, and 76 per arm for post-surgery (subtotal of patients in RCT is 406) (Figure 1) (for a detailed explanation, see Appendix 1).

### Data analysis

Primary analyses will be performed according to intention to treat (ITT) principle. Analyses will be performed with R in R Studio (version 2022.2.1.461; R Foundation for Statistical Computing, Vienna, Austria) and uses P < 0.05 to indicate statistical significance.

#### Effect evaluation primary outcome parameter

To evaluate the difference in clinical effectiveness of both follow-up protocols, the number of radiographs with treatment consequences will be divided by the total number of radiographs in each subgroup (pre-treatment, post-brace, post-surgery). To compare the primary outcome between the PE-FU group and the S-FU group, Pearson’s chi-square test is used. To evaluate the effect according to subgroups, linear mixed-effects models that account for repeated measurements will be performed, adjusted for scoliosis center and relevant baseline covariates. Assumptions of linear mixed modelling will be assessed and if needed variable transformations will be performed. Furthermore, for each subgroup the results of the RCT follow-up protocols will be compared with the similar preference cohort to indicate generalizability of study results.

#### Effect evaluation secondary outcome parameters

Differences in secondary outcome parameters will be compared per subgroup using a similar mixed-effects model, adjusted for relevant covariates, to that used for the primary outcome. The relative contribution of preference for intervention or comparator on the primary outcome will be examined using stepwise logistic regression on all baseline parameters. Similar models will be built to examine the associations of PROMs, Scolioscope, Bunnell scoliometer, changes in radiographic parameters, clinical suspicion of curve progression or patient-reported postoperative complications, number of appointments at outpatient clinic, and number of radiographs with the primary outcome.

#### Economic evaluation

An economic evaluation will be performed per subgroup for quality-adjusted life years (QALYs), treatment consequence (primary outcome), the number of radiographs (secondary outcome), and condition-specific quality of life questionnaire (SRS22r). Missing data will be imputed using multivariate imputation by chained equations and pooled estimates will be calculated using Rubin’s rules [16]. Cost and effect differences will be estimated using seemingly unrelated regression (SUR) analyses or mixed models, depending on the degree of clustering of data [17,18]. Incremental cost-effectiveness ratios (ICERs) will be calculated by dividing the differences in costs by those in effects. Bias-corrected and accelerated bootstrapping with 5,000 replications will be used to estimate the uncertainty surrounding ICERs and 95% confidence intervals around cost differences. Uncertainty will be shown by plotting cost-effectiveness planes and cost-effectiveness acceptability curves. Sensitivity analyses will be performed to assess the robustness of the results.

### Registration, ethics, data management, funding, and disclosures

Prior to the start of inclusion, the study was registered at clinicaltrials.gov (NCT05379127). Ethical approval was obtained by the Medical Ethics Committee (METC; NL77456.091.21). The study will be conducted according to the principles of the Declaration of Helsinki(19) and in accordance with the Medical Research Involving Human Subjects Act (WMO).

In all participating centers, the study protocol will be approved by the local research ethics board. Any substantial amendments will be notified to the accredited METC.

Data will be managed and archived for 15 years at the initiating center (Radboud University Medical Center). For data sharing and management, we intend to comply with the FAIR principles (Findability, Accessibility, Interoperability, and Reusable). All included patients receive a unique trial code, which pseudonymizes their personal data. All data will be coded, stored, and archived following the rules for good clinical practice (GCP). Handling of personal data will comply with the general data protection regulation (GDPR). The outcome data is only accessible for authorized research personnel of the research team at the initiating center, and monitoring and quality assurance personnel. The results from the study will be submitted for publication in peer-reviewed journals and presented at international conferences. This trial is supported by the Dutch Organization for Health Research and Development (ZonMw project number: 10330022010004). In addition to having received the research grant to perform the study, one author has a conflict of interest regarding the Scolioscope. Complete disclosure of interest forms according to ICMJE are available on the article page, doi: 10.2340/17453674.2024.40904

### Steering committee and quality assurance

A steering committee, including an orthopedic spine surgeon, an independent orthopedic surgeon, epidemiologist, patient representative, health technology assessment (HTA) expert, statistician, and implementation specialist, is supported by 8 orthopedic spine surgeons (region coordinators) to ensure acceptance of the study in the AIS consortium. An independent trial bureau (Trialbureau Zorgevaluatie Nederland) has been appointed to assure the quality of the study and to perform data monitoring. Monitoring will be performed in compliance with good clinical practice (GCP), in order to achieve high-quality research and secure patient safety.

## Discussion

This is the largest national and multicenter pragmatic randomized trial addressing the optimal frequency of radiographic follow-up in patients with AIS. It has the following strengths: it is the first study that includes patient-empowered follow-up for AIS, is an inclusive study with 3 follow-up-subgroups and few exclusion criteria, and has preference cohorts alongside the RCT to amplify generalizability. The study is designed as a nationwide multicenter partially randomized preference trial to compare PE-FU with S-FU to avoid unnecessary radiographs in the follow-up of patients with AIS. Patient follow-up is currently based on consensus-based guidelines [4,5]. To our knowledge, this study is the first to compare routine radiographic follow-up with a new patient empowered protocol for follow-up of patients with AIS. This will contribute to the development of evidence-based guidelines.

As an increased incidence of cancer has been reported later in life for patients with AIS receiving radiological follow-up during childhood, this study is relevant for all phases of AIS treatment described in this project [6,7], as the expected number of radiographs will be reduced in the new PE-FU protocol. Standard frequency of radiological follow-up is different in each of the subgroups (pre-treatment, post-brace, and post-surgery). For each subgroup, different indications for a radiograph could exist. This means that the different study objectives will be studied for each subgroup separately and the sample size analysis of each RCT in this study will be performed accordingly.

### Strengths

First, a strength of this study is the preference cohorts alongside the RCTs for each follow-up subgroup. By including preference cohorts, which follows clinical practice, generalizability of study results could be studied while the internal validity is assured and selection bias is reduced. The addition of the preference cohorts also supports implementation of trial results [20]. This design fits with the preferences of the patient panel involved in the grant application for this project [21]. An example of this design is the BrAIST -trial (brace study among patients with AIS) that started as an RCT and ended as an RCT combined with patient preference cohorts. During this trial the design was adapted and, by including a preference cohort, both the randomized groups and the preference cohorts could be completed [20].

Second, the expected risks from participation in the intended study are considered negligible. No risks are associated with the tools used in the study (PROMs, Bunnell scoliometer, Scoliscoop), because these tools are used within the intended indication. For the pre-treatment and post-brace subgroups, a potential but limited risk might exist. The detection of curve progression might be delayed and, consequently, the subsequent initiation of treatment. Due to safety and ethical considerations and to minimize this risk of late detection, the patients who participate in the new PE-FU will still come to the hospital for clinical examination. In addition, to achieve high-quality research and secure patient safety, Trialbureau Zorgevaluatie Nederland is involved in this study to give support in study logistics and to perform the monitoring.

### Limitations

First, the clinically relevant cut-off points for so-called “sense of alarm” of the existing PROMS for AIS follow-up are unknown. This is a secondary outcome of the study and has been accounted for by pre-defining “sense of alarm” criteria. Secondary analyses are planned to determine the relevant sense of alarm criteria. Second is the logistic complexity of the study. Due to this complexity a risk of delay in recruitment might exist. Regular meetings (face-to-face and online) with the local study teams will be planned, to start the study, to discuss possible solutions on the different (logistic) challenges that emerge, and thus to support patient recruitment.

## Notes

JTFB: writing original draft manuscript, revision of manuscript, and project administration. MLvH: supervision, co-principal investigator, review, editing, methodology, and statistical support; PB, JPHJR, TPCS, HMvW: review, editing, investigation, and methodology; JMvD: review, editing, methodology, and statistical support; DGvdF: review, editing, and project administration; EH, PCW: review, editing, and investigation; DHRK: review, editing, investigation, methodology, and statistical support; PJvdW: review, editing, methodology, and statistical support; MdK: review, principal investigator, and methodology. All authors contributed to the protocol and read and approved the final manuscript.

Handling co-editors: Paul Gerdhem and Philippe Wagner

*Acta* thanks Thomas Johan Kibsgård and Acke Ohlin for help with peer review of this manuscript.

## Appendix

### Detailed explanation of sample size calculation

The sample size is based on identifying superiority of the PE-FU in each individual RCT. The sample size is determined to have sufficient power to detect clinically relevant progression of the deformity leading to a treatment consequence. This partially randomized preference trial consists of 3 RCTs and 3 prospective preference cohorts for each treatment arm. The 3 subgroups for this study differ in nature, and as such we calculated the required separate sample sizes for each of the 3 RCTs. Our previous survey of patients with AIS showed that 50% of patients had a preference for 1 of the 2 cohorts (the preference cohorts) and 50% were willing to participate in the RCT. Thus, by doubling the sample size needed for the RCTs, the total sample size is achieved (RCTs and preference cohorts). As no previous studies regarding this subject are available, we searched the literature for patient samples with AIS that resemble our study population and that showed progression of the curve during natural history [14] or after surgery [15]. Assumptions were made based on the literature and expert opinion. Typically, patients with AIS and their caregivers/parents are very compliant with follow-up. Although complete adherence to the follow-up protocols of both the comparator (S-FU) and PE-FU is expected, a 10% dropout or lost-to-FU for the 3 subgroups is expected.

*Usual/S-FU care (comparator):* Although large practice variation exists, a recent survey showed that the average follow-up interval for routine radiographs was 6 months for the pre-treatment and post-brace group and 3 months for the post-surgery group. For all 3 groups biplanar full spine radiographs (PA and lateral) will be obtained at baseline, uniplanar full spine radiographs (PA) at follow-up for the pre-treatment and post-brace group, and biplanar full spine radiographs for the post-surgical group will be obtained. Consensus and acceptance was obtained among the scoliosis centers to define this as usual/S-FU care. In summary:

For the pre-treatment and post-brace group this means:

- Biplanar full spine radiographs (PA and lateral) at intake (baseline assessment study).
- Uniplanar full spine radiographs (PA) at 6, 12, 18 months’ follow-up.
- Biplanar full spine radiographs (PA and lateral) at 24 months’ follow-up at the end point of study (study purpose).
- In total, 2 biplanar and 3 uniplanar full spine radiographs (i.e., 5 assessments with 7 full spine radiographs).
- For the postoperative group this means:
- Biplanar full spine radiographs (PA and lateral) directly postoperatively (baseline assessment study), and at 3 and 12 months’ follow-up.
- Biplanar full spine radiographs (PA and lateral) at 24 months’ follow-up at the end point of study (usual care).

In total, 4 biplanar full spine radiographs (i.e., 4 assessments with 8 full spine radiographs)

### Explanation of the sample size calculation per subgroup

#### Pre-treatment group

In the literature, a 23% risk of curve progression was described [25] (i.e., comparator; usual/S-FU care). In the pre-treatment group, routine follow-up takes place every 6 months for a duration of 24 months (i.e., 4 follow-up assessments). Consequently, the incidence of progression per radiograph is 5.75% (i.e., 23%/4) for the control group/S-FU group. Based on the literature and expert opinion, a 5% margin is allowed for the PE-FU and 20% extra radiographs are expected for this group. The margin of 5% results in 28% risk of curve progression for the investigational group/PE-FU group (23% + 5% = 28%). Therefore, the incidence of progression per radiograph is expected to be 23.3% (i.e., 28%/1.20) for the PE-FU group (intervention/investigational group). For the increase in incidence/radiograph from 5.75% to 23.3%, the calculated sample size is 60 patients per arm (given a 95% CI and 80% power). Taking into account the expected loss to follow-up/dropout (10%), the final sample size is 66 patients for each treatment arm in the pre-treatment group.

#### Post-brace group

In this group also, 23% of the patients have a risk of curve progression [14] (i.e., comparator; usual/S-FU care). The routine follow-up takes place every 6 months for a duration of 24 months (i.e., 4 follow-up assessments). Therefore, the incidence of progression per radiograph is 5.75% (i.e., 23%/4) for the control group/S-FU group. With a 5% margin for the PE-FU group, the risk of curve progression for the investigational group/PE-FU group will be 28%. We expected an increase of 15% based on expert opinion in the number of radiographs in the PE-FU post-brace group. Consequently, the expected incidence of progression per radiograph is 24.3% (i.e., 28%/1.15) in the PE-FU group. Based on the increase in incidence per radiograph from 5.75% to 24.3%, each arm of the post-brace group was calculated to consist of 55 patients (given a 95% CI and 80% power). With an expected loss to follow-up/dropout of 10%, each treatment arm of the post-brace group includes 61 patients.

#### Post-surgery group

Based on a recent study among patients with AIS, no progression in the surgical area is expected [31]. Adding-on is a radiological complication that indicates progression of the curve under or above the instrumented curve. The expert opinion for clinically relevant prevalence of adding-on is 10% (i.e., comparator; usual care), with a 5% margin for PE-FU (i.e., 15%). The routine follow-up takes place at 3, 12, and 24 months after surgery (i.e., 3 follow-up assessments with biplanar full spine radiographs = 6 radiographs). The incidence of adding-on per radiograph is 1.7% (i.e., 10%/6) for the control group/S-FU group. The risk of adding-on for the investigational group/PE-FU group will be 15% (prevalence of 10% with a 5% margin for PE-FU). The expected extra number of radiographs in the post-surgery group is 5% (expert opinion). Therefore, the incidence of adding on per radiograph is expected to be 14.3% (i.e., 15%/1.05). For the increase in incidence/radiograph from 1.7% to 14.3%, the calculated sample size is 69 patients per arm (given a 95% CI and 80% power). Considering a 10% dropout and/or loss to follow-up, the final sample size is 76 patients per treatment arm for the post-surgery group.

Based on the above (literature and expert opinion), for this study a total of 812 patients is required (alpha 0.05; power 0.80). This means 66 per arm for the RCT pre-treatment, 61 per arm for the RCT post-brace, and for the RCT post-surgery 76 per arm (subtotal of patients in the RCTs is 406). The preference cohorts consist of the same arms as the three RCTs. For each arm (preference cohort) a similar number of patients is required (subtotal of patients in the preference cohorts is 406 patients).
